# Effects of biotic and abiotic factors on resistance versus resilience of Douglas fir to drought

**DOI:** 10.1371/journal.pone.0185604

**Published:** 2017-10-03

**Authors:** Gunnar Carnwath, Cara Nelson

**Affiliations:** Department of Ecosystem and Conservation Sciences, W.A. Franke College of Forestry and Conservation, University of Montana, Missoula, Montana, United States of America; Pacific Northwest National Laboratory, UNITED STATES

## Abstract

Significant increases in tree mortality due to drought-induced physiological stress have been documented worldwide. This trend is likely to continue with increased frequency and severity of extreme drought events in the future. Therefore, understanding the factors that influence variability in drought responses among trees will be critical to predicting ecosystem responses to climate change and developing effective management actions. In this study, we used hierarchical mixed-effects models to analyze drought responses of *Pseudotsuga menziesii* in 20 unmanaged forests stands across a broad range of environmental conditions in northeastern Washington, USA. We aimed to 1) identify the biotic and abiotic attributes most closely associated with the responses of individual trees to drought and 2) quantify the variability in drought responses at different spatial scales. We found that growth rates and competition for resources significantly affected resistance to a severe drought event in 2001: slow-growing trees and trees growing in subordinate canopy positions and/or with more neighbors suffered greater declines in radial growth during the drought event. In contrast, the ability of a tree to return to normal growth when climatic conditions improved (resilience) was unaffected by competition or relative growth rates. Drought responses were significantly influenced by tree age: older trees were more resistant but less resilient than younger trees. Finally, we found differences between resistance and resilience in spatial scale: a significant proportion (approximately 50%) of the variability in drought resistance across the study area was at broad spatial scales (i.e. among different forest types), most likely due to differences in the total amount of precipitation received at different elevations; in contrast, variation in resilience was overwhelmingly (82%) at the level of individual trees within stands and there was no difference in drought resilience among forest types. Our results suggest that for *Pseudotsuga menziesii* resistance and resilience to drought are driven by different factors and vary at different spatial scales.

## Introduction

During the last 40 years, there have been significant global increases in the intensity and duration of droughts, and current climate models predict this trend will continue in the future [1]. Although vegetation responses to periodic water stress is an important structuring force across multiple biological scales [2], recent research has underscored the potential for extreme drought events to push ecosystems beyond stability thresholds [3]. Numerous studies across a range of forest types have reported regional, drought-induced mortality of overstory trees [4–6] with cascading effects ranging from changes in phenology of understory vegetation [7] to food web disruption [8] and even to major shifts in ecosystem carbon cycling [9, 10]. However, the effects of extreme events on tree mortality likely are not uniform but rather vary significantly both at large (e.g., within a region and among forest types) and small (among individuals of the same species in the same population) spatial scales [11]. Therefore, understanding the key factors influencing variability in drought responses within species and across sites will be critical for accurately predicting vegetation responses to climate change and developing effective management actions that enhance ecosystem stability.

Drought stress occurs when soil water content is so low that trees can no longer maintain normal life processes. Physiological responses to drought vary as a function of the relative decrease in water availability (drought intensity) and the length of the event (drought duration) (reviewed by [12]). In the short term, trees can minimize water lost through transpiration by closing stomata. If a drought is sufficiently intense, however, high evaporative demand coupled with low soil water availability leads to extreme tension in the xylem and, potentially, to hydraulic failure and desiccation of living tissues. During periods of prolonged water stress, trees may begin to shed leaves and shift allocation of resources from leaves to roots and sapwood. Although these physiological responses buffer xylem tensions and minimize risk of cavitation, they may also have longer-term consequences, including reduced carbon assimilation and growth [13]. Consequently, trees often exhibit the effects of extreme climatic events for several years after they occur [14], and drought-induced mortality can lag anywhere from years to decades following extreme droughts [15].

The physiological consequences of water stress also vary with stand- and tree-level factors. For instance, within a species, drought-induced mortality of overstory trees can vary substantially among stands of different forest types [16] as well as among trees within a stand [17, 18]. At the tree level, numerous studies have shown that the effect of water stress on tree growth varies with tree size and age [19, 20]. This may be related to shifts in carbon allocation associated with ageing [21] or to increasingly negative water potentials associated with longer path lengths as trees reach their maximum size [22].

In addition, long-term stressors such as competition may also “weaken” a tree and reduce its resistance to short-term inciting factors, including extreme drought events [23, 24]. Linares et al. [25], for example, found that *Abies pinsapo* with high levels of competition suffered greater growth declines during dry periods and suggested that the interacting effects of competition and drought contribute to drought-induced mortality. However, suppressed trees are also exposed to substantially different environmental conditions compared to dominant trees, including lower wind velocity, temperature and vapor pressure deficit [26]. These factors have direct and immediate impacts on transpiration rates and have been shown to ameliorate the negative effects of particularly intense or prolonged drought events [27, 28].

It is well established that trees growing on xeric sites are more sensitive to annual fluctuations in water availability than are those growing in cool, moist forests or in sheltered conditions [29]. There is also ample evidence that stand-level differences in aspect [30–32], elevation [33–35], and latitude [36, 37] can significantly affect mean climate responses of mature trees. There is reason to believe that these general climate-growth relationships may not hold under extreme conditions [6, 38]. However, the relative impact of extreme climatic conditions on trees growing on contrasting sites is not well understood. Previous studies of the effects of physical site conditions on drought responses have produced variable, even contradictory, results. For example, radial growth of *Thuja occidentalis* was more affected by drought when growing in xeric sites than when growing in mesic sites [39], but Orwig and Abrams [40] found the opposite results for *Pinus virgniana*. Similarly, in separate studies of *Pinus edulis* in northern Arizona, Ogle et al. [41] found that soil type was an important predictor of mortality following a severe drought in 1996, but Koepke et al. [42] reached the opposite conclusion following a drought in the same region in 2002, suggesting a possible interaction between site conditions and drought duration and/or the timing of drought events. Stand-level variability in abiotic factors (soils, elevation, slope, aspect) may also interact with biotic factors, like stand structure and composition [16](e.g. [43]) or site—specific differences in disturbance history (e.g. [44]), leading to patchiness and spatial complexity in drought responses at the stand or forest level.

Ecological stability—the tendency of an ecosystem, population or individual to return to equilibrium following environmental disturbance or stress—has been described as a function of two related characteristics: *resistance* (the degree of response to a perturbation) and *resilience* (the ability of a system to return to its former state; i.e. engineering resilience [45])(Pimm 1984). It is becoming increasingly clear that factors influencing the resistance and resilience of trees to extreme drought events are complex, operate at multiple scales, and interact in ways difficult to predict [46]. Resistance and resilience of ecological systems are generally estimated by comparing performance of organisms responding to stress to control organisms not experiencing the stress [47, 48]. Using tree-rings, a stress response can be accurately measured, compared to growth under baseline conditions and then simultaneously analyzed across space and time to reveal and disentangle the key environmental factors that regulate drought responses [44, 49–51]. In the present study, we analyzed tree-ring width series of *Pseudotsuga menziesii* from 20 stands across a broad range of environmental conditions and assessed variation in responses of individual trees to a severe drought event in 2001. Our primary objectives were to 1) identify the specific biotic and abiotic attributes that were most closely associated with ecological stability (i.e. resistance and resilience as defined above); and 2) assess the variability in drought responses at different spatial scales. A better understanding of these dynamics is crucial to accurately predicting tree and stand-level responses to climate change.

## Methods

### Study area and site selection

**Fig 1:**
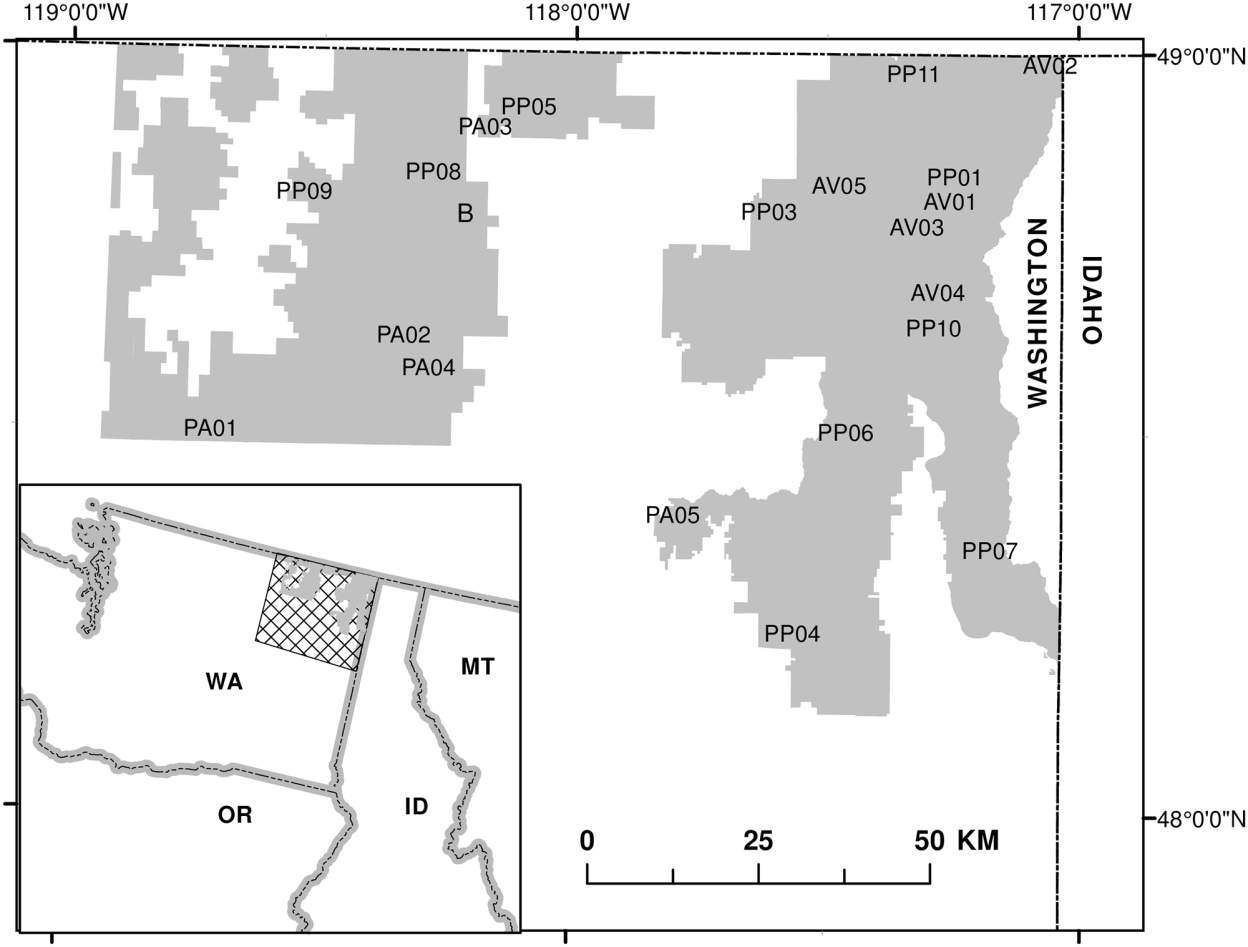
Location of study sites. Colville National Forest (darker shaded area) in northeastern Washington State (inset). Capital letters of the Stand ID indicate the forest type [PA = *Pinus ponderosa-Pseudotsuga menziesii/Agropyron spicatum*; PP = *Pseudotsuga menziesii/Physocarpus malcaeus*, AV = *Abies lasiocarpa/Vaccinium membranaceum*]. The numbers indicate the stand number within that forest type.

This study was conducted on the Colville National Forest (CNF) in northeastern Washington between 48°N and 49°N latitude and 117°W and 119°W longitude (Fig 1). Permission for all field work was given by the CNF.

With a range of 30 to 135 cm precipitation per year, the west side of the CNF is strongly influenced by a rain shadow formed by the northern Cascades, while the northeastern region has a near-maritime climate due to a westerly airflow forced over the Selkirk and Kettle River mountain ranges. To capture the variation in drought responses of Douglas fir at different spatial scales, we used a multi-level sampling design in which individual trees (the sampling unit) were nested within stands, which were then further nested within distinct forest types. At the broadest scale, sampling was stratified by the Forested Plant Association Group (PAG) [52]. PAGs separate distinct biophysical environments based on shared floristics, environment and productivity and are a central component of commonly used vegetation models, including recent efforts attempting to link effects of climate change with project-level planning (e.g. FVS-CLIM [53]).

Three PAGs (hereafter “forest type”) were selected for sampling: 1) *Pinus ponderosa-Pseudotsuga menziesii/Agropyron spicatum* (hereafter “PIPO”); 2) *Pseudotsuga menziesii/Physocarpus malcaeus* (hereafter “PSME”); 3) *Abies lasiocarpa/Vaccinium membranaceum* (hereafter “ABLA”). PIPO is the hottest and driest forest type in the CNF and generally occurs below 1,000 m; vegetation is characterized by open stands of ponderosa pine and Douglas fir, with a bunch-grass-dominated understory and few shrubs. PSME is the most common forest type in this region. It is generally cooler and wetter than PIPO and occurs at a broad range in elevation (approximately 500–1,500 m). *Physocarpus malvaceus* and *Holodiscus discolour* are the most prevalent shrubs, but *Amelanchier arborea* and *Mahonia aquifolium* are also quite common. Douglas fir is the most common tree species and ponderosa pine is a major seral species. The wettest forest type, ABLA, is well distributed across the study area at elevations above 1,500 m and includes upland forest stands with either *Abies lasiocarpa* or *Picea engelmannii* as the climax species.

Within each forest type, we used a GIS to identify stands with the following criteria: 1) minimum size of 8 ha; 2) southwest-southeast aspect; 3) mid-slope position on an approximately 40% slope; and 4) no significant disturbance (such as logging or fire) in the last 60 years. Prior to sampling, all potential sites were visited to see that these conditions were met and to verify that there was no evidence of pathogenic outbreaks, substantial mistletoe or windthrow. Through this process, we identified a total of 20 suitable sites broadly distributed across the study area (Table 1, Fig 1). PIPO sites were an average of approximately 200 m lower in elevation than PSME sites and 600 lower than ABLA sites. From 1950–2007, the average annual precipitation ranged from 47 mm in PIPO stands to 60 mm and 121 mm in PSME and ABLA sites, while average annual temperatures were 6.6°C, 5.9°C and 3.9°C respectively.

**Table 1:** Study site summary information.

Stand ID	Forest Type	Latitude	Longitude	Elevation (m)	BA (m^2^/ha)	Site index	Precip. (mm)	Temp. (°C)	# Trees	Mean age
AV01	ABLA	48.80	-117.254	1748	51.2	41	111	4.0	14	156
AV02	ABLA	48.99	-117.058	1653	49.4	48	150	2.1	19	187
AV03	ABLA	48.77	-117.322	1613	45.0	48	101	5.5	15	111
AV04	ABLA	48.69	-117.279	1592	31.7	50	142	3.1	20	67
AV05	ABLA	48.83	-117.477	1561	45.9	50	101	4.7	17	72
PA01	PIPO	48.50	-118.713	957	31.4	41	33	5.5	29	95
PA02	PIPO	48.82	-118.211	651	27.1	57	43	7.3	30	88
PA03	PIPO	48.90	-118.181	976	13.1	37	51	7.5	24	97
PA04	PIPO	48.58	-118.285	933	23.4	68	45	5.5	24	88
PA05	PSME	48.39	-117.799	1069	25.0	51	61	7.2	27	90
PP01	PSME	48.84	-117.245	1097	33.7	42	77	4.4	28	153
PP02	PSME	48.79	-117.615	1158	38.3	56	67	7.1	26	102
PP03	PSME	48.24	-117.563	1128	41.3	65	60	6.1	29	78
PP04	PSME	48.91	-118.153	914	34.4	51	51	7.5	25	119
PP05	PSME	48.61	-118.307	1250	45.9	74	59	4.9	27	88
PP06	PSME	48.35	-117.175	1250	40.9	60	80	6.2	26	90
PP07	PSME	48.84	-118.282	1311	34.0	55	52	5.1	26	120
PP08	PSME	48.81	-118.538	1128	40.9	59	39	5.2	22	127
PP09	PSME	48.64	-117.288	975	35.4	64	62	6.1	25	83
PP10	PSME	48.98	-117.329	884	34.9	52	55	6.7	28	95

For each sampled stand, forest type, geographic location (degrees latitude and longitude), elevation, basal area, site index, average annual precipitation and temperature, number of trees sampled (# Trees), and mean tree age.

### Sampling methods

From each stand, we sampled 10–15 dominant/co-dominant trees (trees receiving full light from above and partial from the sides; hereafter “dominant”) and 10–15 intermediate trees (trees in definitively subordinate positions, receiving little direct light from above and no light from the sides; hereafter “intermediate”). Although the PIPO stands were generally less dense than either the ABLA or PSME forest types, trees often occur in dense clumps containing numerous intermediate trees to sample at each site. Fewer intermediate trees were sampled in ABLA stands because Douglas fir generally occurs as seral remnants and individuals in sub-dominant canopy positions were relatively scarce in this plant association. Trees selected for sampling met the following criteria: 1) no obvious defects such as cankers, scars, rot, substantial lean or mistletoe infestation; 2) >50 years old at breast height (1.3 m); 3) >50 m from the edge of the stand and other sampled trees of the same canopy class; 4) >10 cm diameter at breast height (DBH); and 5) >10 m from any dead or dying trees.

For each subject tree, tree height was measured using a laser hypsometer, and crown width was estimated as the average length from the stem to the tip of the longest branch at each of the four cardinal directions. Abundance of understory vegetation was estimated within the drip line using four wedge-shaped subplots. In each subplot, abundance was estimated separately for herbs and shrubs using broad percent-cover categories (0%, 1–25%, 26–50%, 51–75%, 76–100%). The zone of influence around each subject tree was identified using a fixed-angle gauge (Basal Area Factor [BAF] = 10 for PIPO sites and 20 for PSME and ABLA sites respectively) [54]. To estimate the basal area (BA) of competitor trees, we multiplied the total number of trees identified in this zone by the BAF.

Site index (SI) is a tool to determine the relative productivity of a particular site or location. SI is the height of a "free to grow" tree of a given species at a base age on the site of interest. SI is strongly correlated with temperature and growing season length and can be strongly affected by climate conditions [53]. Site index was estimated using all dominant and co-dominant trees from each site according to the method described by Monserud (1984) [55] for inland Douglas fir.

### Dendrochronological methods

For each sampled tree, we extracted two cores with an increment borer at breast height from opposite sides of the stem and perpendicular to the fall line of the slope. Cores were transported to the lab in protective straws, mounted in wooden mounts, and sanded with progressively finer sandpaper using standard techniques [29, 56]. We visually cross-dated all cores and calculated tree age by counting annual rings. When the pith was absent from increment cores, we used a concentric ring pith locator to estimate age [57]. Increment cores were scanned using an optical scanner at 1200 dpi resolution and all rings of each core were measured to the nearest 0.001 mm using the CooRecorder software [58]. Occasionally, the image resolution was inadequate to confidently measure the smallest rings. In these cases, cores were measured using a microscope and a Velmex sliding stage micrometer interfaced with a computer. We checked for cross-dating errors with the software programs COFECHA [59] and CDendro 7.1 [60]. Only cores that could be confidently cross-dated were statistically analyzed. Finally, we averaged the two tree-ring width measurements from the same tree by year to produce one mean ring-width time series for each sampled tree.

Basal area (BA) of each subject tree was calculated by assuming a circular cross section and using inside-bark radius (*BA* = *πr*^2^). Bark thickness was calculated according to the formula developed for interior Douglas fir by Monserud and Forest [61]. With this information, a relative growth rate (RGR) was then calculated for each tree as the ratio of total radial growth (cm^2^) from 1998 to 2007 (BAI_10_) to tree size in 1998 (RGR = *BAI*_10_/*BA*–*BAI*_10_).

### Climate data

The Parameter-elevation Relationships on Independent Slopes Model (PRISM) dataset [62] was used to obtain fine-scale (800m) gridded monthly precipitation and temperature climate data for each sampled stand. The PRISM model weighs individual climate station data and estimates values across a landscape accounting for differences in elevation, aspect, and topographic exposure.

Regional water stress was estimated using Palmer drought severity index (PDSI) (Alley, 1984) data obtained from the National Climate Data Center for Climate (NCDC) Division 9 of Washington state (northeastern Washington, available at http://www.ncdc.noaa.gov). The PDSI scale is centered on zero with negative numbers indicating drier than average conditions; values less than negative 3 are classified as “severe” drought, while negative 4 is considered “extreme” drought.

### Quantifying drought responses

Ratios of growth during and after drought years to growth during an “average” year are commonly used to quantify the impact to drought and the subsequent recovery [14, 44, 50, 63]. Here, to estimate expected growth in the absence of drought disturbance (i.e. the baseline), we fit a curve to each tree-ring series using cubic splines with a 50% frequency-response cut-off at 30-year periods [64]. This commonly used method, known as “detrending” produces a curve that represents a tree’s predicted annual growth increment, that accounts for low-frequency biological growth trends (related to changes in tree age and size) without filtering out effects of climate variability.

To quantify the degree of departure from predicted growth in a given year (i.e. resistance and resilience), we computed the ratio between measured ring-widths and the corresponding fitted values. This resulted in a dimensionless ring-width index (RWI) that has numerous advantages over using raw data including removing differences in ring widths related to variability in tree size or age, and rescaling each series to a mean of one and near constant variance [29]. RWI values > 1 indicate above average growth while RWI < 1 denotes below average growth.

**Fig 2:**
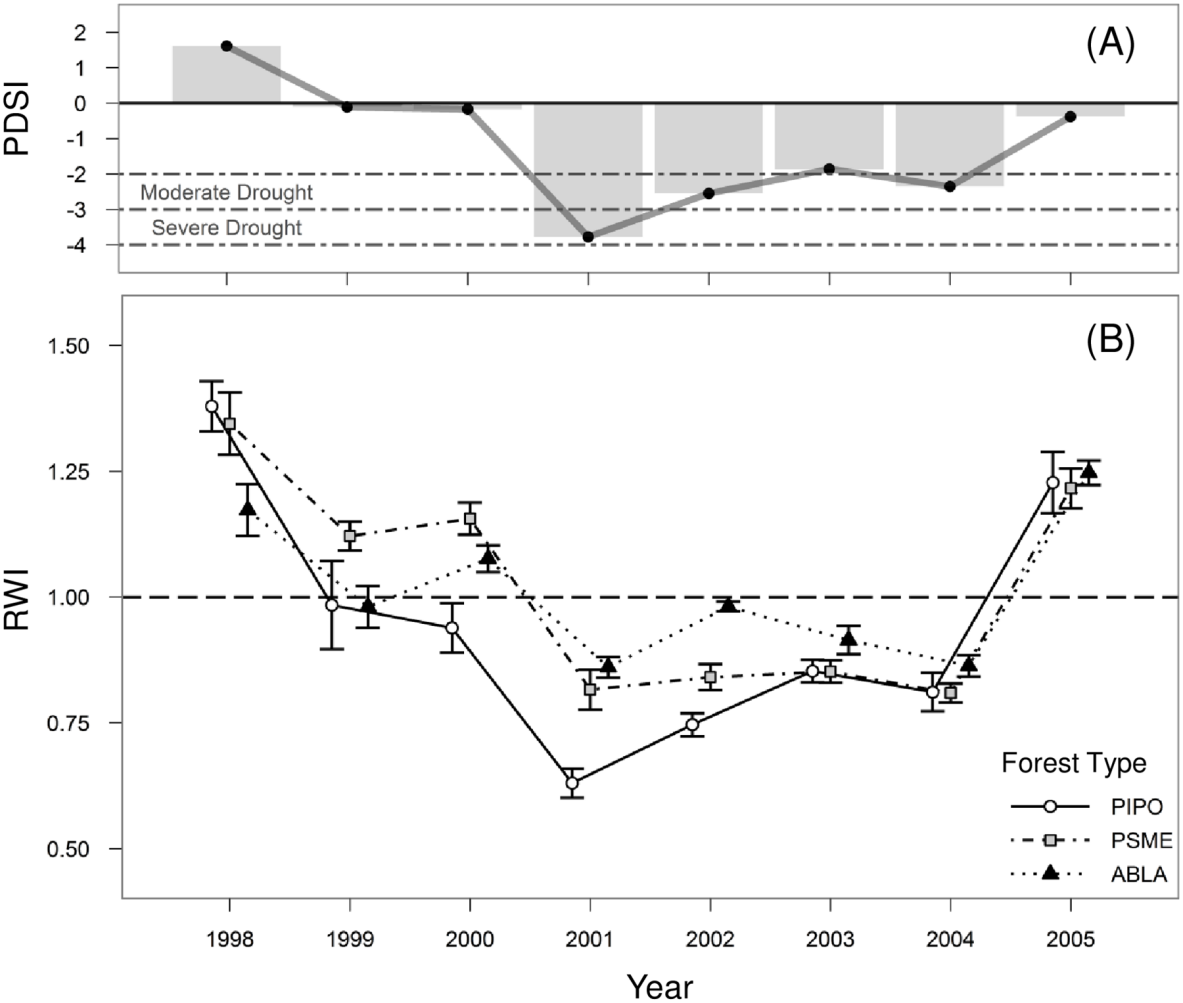
Time series showing drought severity and corresponding tree growth from 1998 to 2005. (A) Annual average Palmer drought severity index (PDSI) for Climate Division 9, Washington. (B) Standardized growth index values (RWI; stand-level mean ± SE) of *Pseudotsuga Menziesii* by forest type [PIPO = *Pinus ponderosa-Pseudotsuga menziesii/Agropyron spicatum* (circles; n = 5), PSME = *Pseudotsuga menziesii/Physocarpus malcaeus* (squares; n = 10), ABLA = *Abies lasiocarpa/Vaccinium membranaceum* (triangles; n = 5). Dashed line indicates average growth (RWI = 1).

We focused on tree responses to a severe drought that occurred in 2001. According to data obtained from the NCDC Division 9, the total precipitation received in the study area during the growing season (May–August) was 9.5 cm; approximately 64% of the long—term (1950–2000) average. Based on PDSI, the 2001 drought event is considered “severe” and the most intense drought in over 30 years. 2001 was also a year of abnormally low growth across the entire study area (Fig 2), suggesting that this disturbance event was driven by regional climate patterns—specifically high temperatures coupled with low precipitation—and not local drivers such as insect outbreaks.

We assessed resistance by comparing tree performance in the year of drought to performance without the effects of drought disturbance (*sensu* Pimm 1984). To assess variability in resistance (i.e. the degree of change in radial growth caused by the disturbance event), we used RWI values in 2001, the severe drought year, as the primary response variable. To assess resilience—defined here as a tree’s ability to return to average growth following a disturbance (*sensu* Pimm 1984)–we analyzed RWI values in 2005, the first year PDSI returned to near 0, indicating that soil moisture had returned to average. In addition, in order to characterize the full curve of recovery, we also analyzed growth responses (RWI) each year from 2002 to 2004 (hereafter “recovery”). These three years were significantly drier than the long-term average (average annual PDSI = -2.5, -1.9 and -2.4 for 2002, 2003, and 2004 respectively). As such, the recovery period is characterized by consistently moderate, though not severe, soil water deficits. Because tree growth in prior years can influence growth in proceeding years, we also included RWI in the two years preceding the focal year (RWI_Lag1 and RWI Lag2, respectively).

### Statistical analysis

To characterize the factors that were most important in determining the drought responses of individual trees, we used multi-level linear mixed effects (LME) models. By explicitly distinguishing between distinct sources of variation—population-averaged (main effects) and group-specific (random effects)–LME models allow for uneven sample sizes and for the covariance of error within groups associated with a nested data structure [65]. As such, mixed-effects models specifically account for the spatial autocorrelation between trees at the same scale, which allows for a more accurate inference of the fixed effects of interest. Using separate models for each year, we modeled drought resistance (RWI in 2001), recovery (RWI in years 2002–2004) and resilience (RWI in 2005) with random effects of forest type and stand (see Table 2 for a full list of fixed effects). Fixed-effect coefficients were estimated using maximum-likelihood (ML) along with the Markov Chain Monte Carlo (MCMC) method [65]. Insignificant variables (at *P* > 0.05) in any model year were removed from the final model. However, if a variable was significant in at least one year, it was retained in the final model for all years to facilitate informal comparison of coefficient estimates between years. An estimate of the total variance explained (R^2^) in each model was calculated using likelihood ratio statistics [66]. Diagnostic plots were used to validate assumptions about residuals and random effects [65]; no deviations from assumptions were detected. Predictor variables were log transformed to improve model fit when needed (see Table 2). Multicollinearity of predictor variables was assessed via the variance inflation factor (VIF); VIF values were low (< 3), indicating low collinearity.

**Table 2:** Variables included in mixed effects models.

Variable Name	Description	Units
**Biotic Factors**	(All tree-level variables measured in 2008 and 2009)	
DBH	Diameter at breast height	cm
Height	Tree height; measured with a laser hypsomter	m
LCR	Live crown ratio, the ratio of the vertical distance from the tip of the leader to the base of the crown (the lowest live whorl) to tree height	**-**
Age	Tree age, estimated from annual rings	years
Canopy	Canopy class, a classification of the position of an individual tree’s crown relative to the rest of the forest canopy; levels = dominant (DO) or intermediate (IN)	
Competition	Basal area of competitor trees, estimated from variable radius plots centered on each subject tree	m^2^ ha^-1^
RWI_Lag1	Ring width index in previous year	**-**
RWI_Lag2	Ring width index two years prior	**-**
Shrubs	Index of shrub cover under dripline of subject tree; levels = 1–5	**-**
Herbs	Index of herbaceous plant cover under dripline of subject tree; levels = 1–5	**-**
CW	Crown width, average span of tree crown	m
RGR	Relative growth rate, calculated as basal area increment from 1998–2007 divided by the subject tree’s basal area in 1998.	
**Abiotic Factors**		
SI	Site index, index of potential productivity of a site based on the height of dominant trees at 50 years	-
ELEV	Elevation	m
TEMP	Average annual temperature in current year, estimated using PRISM climate data	°C
PRCP	Total precipitation in current year, estimated using PRISM climate data	mm

We used the random effect components to assess the proportion of the variability in drought responses at each scale of the sampling [65]. The proportion of the total variance associated with (forest type), stand and individual trees was calculated and converted to a percentage. We fit the models for each year using restricted maximum-likelihood and then tested the significance of random effects using likelihood ratio tests.

All calculations and analyses were accomplished with the statistical software R (v 2.14, R Foundation for Statistical Modeling) along with the packages *dplR* for detrending tree-ring series and *nlme* for LME modeling. Data used in this study is freely available at https://figshare.com/s/b988126c8fc5d134d3c9.

## Results

In 2000, the year before the drought, average RWI across all three forest types was 1.06, indicating that mean growth rates were slightly above average in the study area. In 2001, mean RWI decreased approximately 30% to 0.77. Trees in the driest forest type, PIPO, had lower mean RWI than trees in ABLA, the highest and wettest forest type (0.63 and 0.86 respectively; Fig 2).

Drought resistance (measured as RWI in 2001) was significantly influenced by five biotic factors and one abiotic factor (Table 3). Growth in the prior year (RWI_Lag1) had a significant positive influence on growth (*t* = 9.45; *P* < 0.001), while basal area of neighboring trees (competition) had a significant negative effect (*t* = -5.02; *P* < 0.001). Trees in subordinate canopy positions had greater growth reductions relative to dominant or co-dominant individuals (*t* = -2.38; *P* = 0.018; Table 3). Older trees and individuals with high relative growth rates were also significantly more drought resistant (*t* = 2.56; *P* = 0.011 and *t* = 2.71; *P* = 0.007 for Age and RGR respectively; Table 3). The only abiotic variable assessed that significantly affected resistance was total precipitation received that year (*t* = 3.44; *P* = 0.004; Table 3).

**Table 3:** Results of linear mixed-effects models of ring width index (RWI).

		*Resistance*				*Recovery*												*Resilience*			
		2001; R^2^ = 0.61				2002; R^2^ = 0.47				2003; R^2^ = 0.30				2004; R^2^ = 0.17				2005; R^2^ = 0.34			
*Fixed effect*	*df*	Value	SE	*t* value	*P* value	Value	SE	*t* value	*P* value	Value	SE	*t* value	*P* value	Value	SE	*t* value	*P* value	Value	SE	*t* value	*P* value
***Biotic***																					
Intercept	455	**0.570**	**0.184**	**3.101**	**0.002**	**0.568**	**0.165**	**3.436**	**0.001**	**0.434**	**0.197**	**2.200**	**0.028**	**0.562**	**0.198**	**2.846**	**0.005**	**1.734**	**0.256**	**6.768**	**<0.001**
RWI_Lag1	455	**0.295**	**0.031**	**9.452**	**<0.001**	**0.320**	**0.038**	**8.382**	**<0.001**	**0.337**	**0.040**	**8.419**	**<0.001**	**0.140**	**0.041**	**3.444**	**0.001**	**0.310**	**0.038**	**8.129**	**<0.001**
RWI_Lag2	455	-0.023	0.031	-0.727	0.468	**0.156**	**0.031**	**5.091**	**<0.001**	**0.149**	**0.041**	**3.593**	**<0.001**	0.052	0.039	1.329	0.184	**-0.178**	**0.040**	**-4.495**	**<0.001**
Competition	455	**-0.054**	**0.011**	**-5.024**	**<0.001**	0.020	0.012	1.708	0.088	-0.005	0.012	-0.402	0.688	-0.008	0.013	-0.646	0.519	-0.008	0.014	-0.586	0.558
Canopy = IN	455	**-0.042**	**0.018**	**-2.379**	**0.018**	-0.003	0.019	-0.138	0.890	**0.050**	**0.020**	**2.512**	**0.012**	-0.035	0.022	-1.596	0.111	0.027	0.024	1.145	0.253
Height	455	-0.001	0.002	-0.714	0.476	-0.001	0.002	-0.346	0.730	**0.005**	**0.002**	**2.936**	**0.003**	0.001	0.002	0.327	0.744	0.002	0.002	0.874	0.383
Age	455	**0.069**	**0.027**	**2.562**	**0.011**	-0.011	0.028	-0.394	0.694	-0.055	0.030	-1.815	0.070	0.032	0.032	0.977	0.329	**-0.089**	**0.035**	**-2.512**	**0.012**
RGR	455	**0.213**	**0.079**	**2.710**	**0.007**	-0.008	0.086	-0.096	0.923	-0.029	0.089	-0.332	0.740	**0.416**	**0.098**	**4.247**	**<0.001**	-0.160	0.106	-1.514	0.131
***Abiotic***																					
Precipitation	15	**0.197**	**0.057**	**3.444**	**0.004**	0.048	0.031	1.530	0.147	-0.039	0.050	-0.780	0.448	0.024	0.035	0.702	0.494	-0.027	0.087	-0.316	0.756
Site Index	15	-0.003	0.002	-1.501	0.154	**-0.003**	**0.001**	**-2.952**	**0.010**	0.003	0.002	1.939	0.071	-0.001	0.002	-0.904	0.380	-0.004	0.003	-1.203	0.248

Results of linear mixed-effects models of ring width index (RWI) in 2001 (severe drought year; resistance), 2002–2004 (moderately dry years; recovery), and 2005 (return to average soil moisture; resilience). Results for insignificant covariates are not shown. See Table 2 for a description of variables. (n = 481 trees from 20 stands)

In the three years following the 2001 drought (drought recovery), the relative effects of both biotic and abiotic variables fluctuated substantially. RWI in the previous year had a significant effect in all years, but its influence was the least pronounced by 2004 (*t* = 3.44; *P* = 0.001). The effect of RWI two years prior (RWI_Lag2) was most important in 2002 (*t* = 5.09; *P* < 0.001, but was also highly significant in 2003 (*t* = 3.59; *P* < 0.001), two years after the drought event. Tree height had a significant positive effect in 2003 (*t* = 2.94; *P* = 0.003) and no significant effect any other year. Similarly, there were no significant differences between canopy classes in 2002 or 2004, but in 2003 the effect of a subordinate canopy position was significantly positive (*t* = 2.51; *P* = 0.012). With regard to abiotic variables, site index was a significant factor in 2002 (*t* = -2.95; *P* = 0.01). In contrast to the year of the severe drought (2001) variability in precipitation among stands did not have a significant effect on variability in RWI during the moderately dry, post-drought period from 2002–2004. By 2003, abiotic factors were no longer significant.

Drought resilience (measured as RWI in 2005, when soil moisture returned to normal) was significantly affected by three biotic factors. RWI_Lag1 had a significant positive influence (*t* = 8.12; *P* < 0.001), and RWI_Lag2 (i.e. growth in 2003) had a significant negative influence (*t* = -4.53; *P* < 0.001) on drought resilience. The only other biotic variable that was significant in 2005 was age (*t* = -2.51; *P* < 0.012). No abiotic factors significantly affected resilience.

The total amount of variation explained by the model was fairly high in 2001 (R^2^ = 0.61) but progressively declined as the dry conditions persisted in the following years (R^2^ = 0.47, 0.30, 0.17 in 2002, 2003 and 2004 respectively; Table 3). When soil moisture recovered in 2005, R^2^ increased to 0.34 (Table 3).

**Fig 3:**
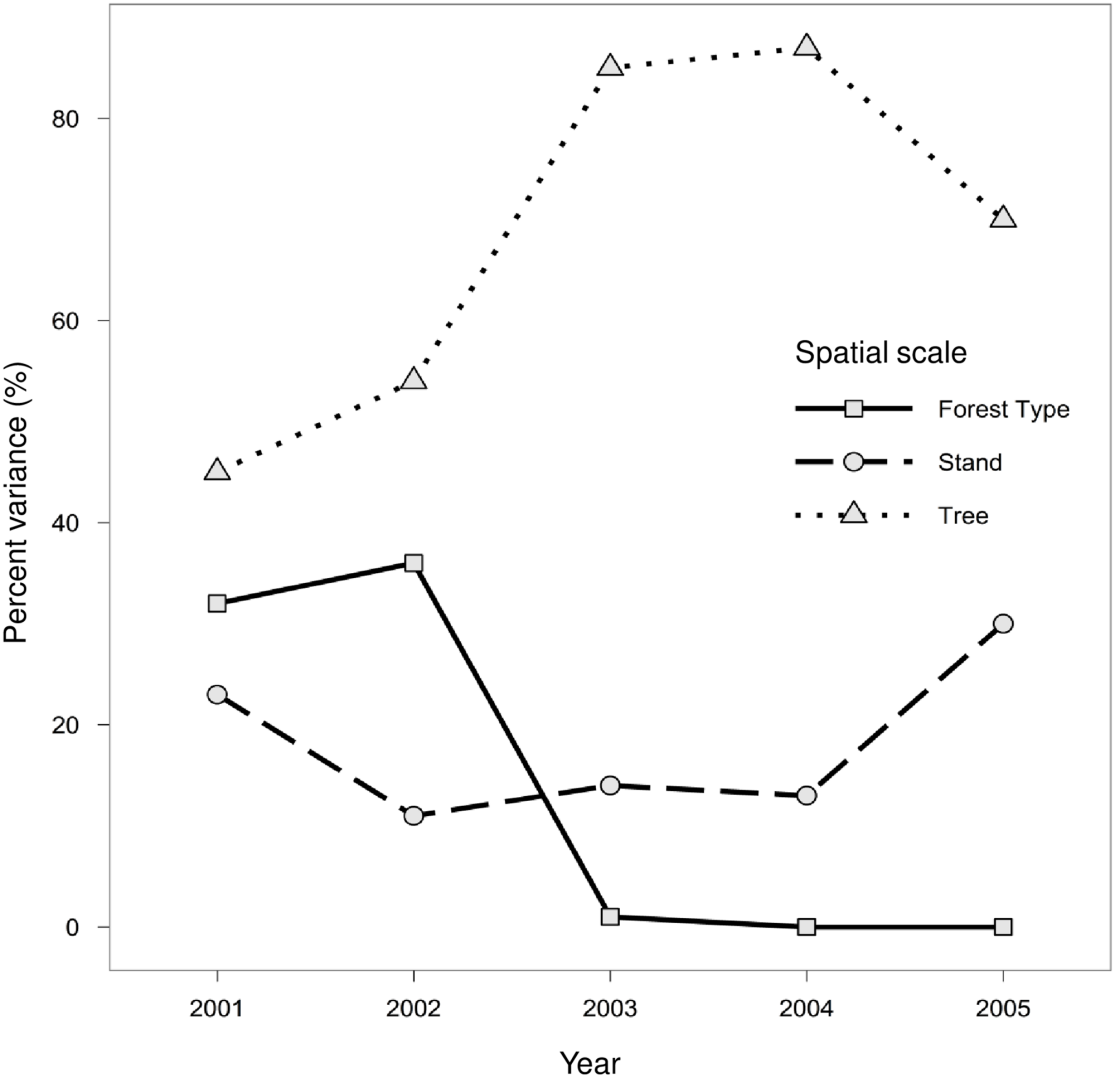
Time series showing change in the proportion of the total variance explained in random effects models. Percent variance shown for each of three nested spatial scales: forest type (squares), stand (circles), and individual trees (triangles).

Thirty-two percent of the overall variance in drought resistance was related to differences among forest types—the broadest spatial scale (L = 6.11; *df* = 1; *P* = 0.014). In 2002, the first drought recovery year, variability at the forest type level increased slightly to 36% (L = 11.35; *df* = 1, *P* = < 0.0001; Fig 3). For the remaining recovery and resilience period, however, variability associated with forest type, was not significant. In contrast, variability at the stand-level accounted for a significant amount of variation in RWI each year. The strongest effect was in 2005 (30% variance; L = 128.69; *df* = 1, *P* < 0.0001; Fig 3), but results were also significant in 2001 through 2004 (34.13<L<111.07, df = 1, *P* < 0.0001; Fig 3). The percent of total variance in RWI was generally highest at the smallest spatial scale, the tree-level. Although it was significant every year, variation associated with among-tree differences changed substantially over time: it was lowest during the drought in 2001 (45%), increased to 54% in 2002 and then to 85%, 87% and 70% in 2003, 2004 and 2005 respectively (Fig 3).

## Discussion

Our study reinforces the idea that ecosystem stability must be considered in terms of at least two distinct components—resistance and resilience—and suggests that these components are likely controlled by different mechanisms that vary at different spatial scales within a landscape. Our primary findings were that 1) competition and relative growth rates affected resistance but not resilience; 2) older trees were more resistant but less resilient than younger trees; and 3) there was a high degree of variability in drought resistance at the broadest spatial scale (forest type) and relatively low variability at the smallest scale (tree-level), while the opposite trend was observed for resilience.

Both the basal area of neighboring trees and the effect of growing in a subordinate canopy position had significant negative parameter estimates, suggesting that trees growing with a high degree of competition suffered the greatest growth reductions during drought. Previous studies investigating the effects of competition on drought responses of conifers have found similar results for *Abies pinsapo* [67], *Pinus strobus* [68], *Picea abies* [69] and *Pinus sylvestris* [70]. Relative to dominants, competitively subordinate Douglas fir trees are known to exhibit significantly reduced rooting depths and a greater shoot-to-root ratio [71]. In the face of sudden severe droughts, these structural limitations lead to lower drought resistance by significantly limiting their ability to acquire scarce resources, including access to deep soil water. This finding is consistent with ecological theory suggesting that when a single resource is limiting, competitive dominance will strongly regulate differences in plant performance [72].

In addition to regulating stomata and shifting biomass allocation from leaves to woody parts, another way individual trees can ameliorate water stress and decrease xylem vulnerability to embolism is by modifying structural properties of their hydraulic architecture and xylem structure [73]. For example, for Douglas-fir, the proportion of latewood in the xylem plays an important role in regulating drought tolerance [74, 75], partly because latewood tracheids are capable of storing significantly more water than are earlywood tracheids [76]. Notably, Douglas fir trees experiencing a high degree of competition have been shown to maintain a higher proportion of sapwood area in latewood compared to trees with few neighbors [28].

Four years post-drought, when climatic conditions returned to normal after a prolonged dry period, growth responses no longer varied as a function of competition or relative growth rates. In addition, the influence of tree age shifted from positive to negative, suggesting that older trees were significantly more drought resistant but less resilient. Previous studies of drought responses in conifers have also found that the primary factors influencing resistance and resilience can differ in both magnitude and direction, but results have been highly variable and difficult to generalize. For example, Lloret et al. (2011) [44]found that fast-growing and younger *Pinus ponderosa* were generally more drought resistant, but older trees recovered better from recent drought events. In a recent study of *pinus sylvestris*, Martínez-Vilalta [77] also found that older trees were less able to recover (less resilient), but, regardless of age, fast growing trees were more severely affected (less resistant). Our finding that fast growing trees were more drought resistant but younger trees were more resilient suggests different mechanisms underlying these two attributes of stability and points toward tree-level changes associated with increasing age or size.

Understanding the key mechanisms and possible tradeoffs between resistance and resilience in long-lived species such as trees is a critical but underdeveloped research area in ecology and climate change science. In an investigation of the relationship between ecosystem stability and biodiversity in conifer forests of the Sierra Nevada, DeClerck et al. [78] found that community resilience, but not resistance, was positively associated with species richness. They suggested that whereas resistance was primarily driven by competition for a limiting resource, resilience is driven by the ability of a community to partition resources in the absence of a single limiting resource. In this study, we also found significant differences in the factors driving resistance and resilience and, consistent with this theory, that trees with a greater ability to acquire resources (i.e. individuals with high relative growth rates and fewer neighbors) were significantly more drought resistant but not necessarily more resilient. Notably, we found that not only did the relative importance of precipitation in the models diminish in the years following the severe drought, but the overall variance explained by the models also dropped significantly. The fact that the variables included in our models provided more predictive power for resistance than for resilience is an interesting finding. Further research is needed to understand the mechanisms underlying this observation and to test the role variables that were beyond the scope of our study (such as community composition or species richness).

This also seems generally consistent with previous research demonstrating greater growth reductions during drought in high-density stands (e.g. [79, 80]) but no relationship between stand density and drought-induced mortality—perhaps the ultimate measure of resilience [16, 18]. It is possible, then, that when water becomes more abundant, a tree’s competitive ability becomes less crucial relative to the partitioning of other resources, such as the relative availability of soil nutrients. Other studies have suggested that tradeoffs between resistance and resilience of conifers may be associated with the production of secondary compounds [81] or the use of stored carbohydrate reserves [82]. However, numerous factors including the role of phenotypic plasticity [75, 83, 84], interactions between drought stress and other pathogens, and high variability in drought responses among species and sites [46] makes it difficult to generalize. Additional research is needed to further describe and disentangle the mechanisms underlying resistance and resilience and to better understand the relationship between drought responses and mortality.

Although the relationship between competition and resistance was significant, the proportion of the variance in RWI at the individual tree-level was lowest in 2001, while variance at the broader spatial scales, particularly the forest type level, was quite high (accounting for more than half the variance in drought resistance). These results counter those of Martínez-Vilalta et al. [77], who found that drought responses of *Pinus sylvestris* were mostly determined by tree-level factors, but that large-scale climatic differences (measured across 393 plots with an 800 m elevation gradient) were relatively unimportant. Here, we found that site-to-site variability in the total amount of precipitation received was a highly significant factor influencing resistance, but not resilience. This suggests that the variation in response among forest types is most likely due to broad-scale gradients in precipitation and water availability. This finding is consistent with the results of Adams and Kolb [85], who showed that the sensitivity of eight tree species to a regional drought event in northern Arizona was significantly related to differences in elevation and consistently greater at the dry end of each species’ regional distribution. Compared to other years, the relatively low proportion of variance at the tree-level during the drought suggests that all trees were strongly affected by the severe drought, regardless of their size or individual growing conditions. Thus, although competition among trees for water was clearly intense in 2001, at the ecosystem-scale it was not very important relative to broad differences in precipitation and water availability: trees growing in cooler, moister forest types suffered the least regardless of competitive status.

Ultimately, a rigorous understanding of tree responses to drought will require careful consideration of several factors not addressed in this study including the role of genetic variability—both within populations and along environmental gradients [86]–as well as a whole tree approach that integrates simultaneous measurements of water and carbon fluxes to make accurate inferences about physiological stress and plant carbon balance]. However, such studies are extremely costly and time intensive; as such, investigations of drought responses at landscape or ecosystem scales will continue to rely on more simplistic analyses of radial growth, particularly tree-ring series. To date, numerous tree-ring based studies, such as those cited above, have made important contributions in this field. However, a meaningful synthesis of results is lacking and problematic, largely because of highly inconsistent tree-ring-based metrics for quantifying drought responses. This barrier stems in part from a lack of agreement on how to define a drought event (particularly the duration of the event), but also reflects a failure to appreciate and explicitly account for how variability in the timing of wet and dry years (both prior to the event and during the recovery stages) could modify drought responses. Here, we used a transparent and straightforward method of quantifying drought responses that avoids the need to designate an arbitrary point in time as *the* dividing point separating resistance from resilience. This approach allowed us to characterize recovery following a drought event and reveal trends that might have been lost in an analysis of growth responses averaged over multiple years. Better and more consistent tree-ring-based metrics for measuring drought resistance and resilience would increase our ability to synthesize results to increase predictive power and better inform future forest management decisions.
